# Discrepancy in Phenological Indicators from CO_2_ Flux, MODIS Image and Ground Observation in a Temperate Mixed Forest and an Alpine Shrub Ecosystem

**DOI:** 10.3390/plants15010039

**Published:** 2025-12-22

**Authors:** Chuying Guo, Leiming Zhang, Peiyu Cao, Wenxing Luo, Rong Huang

**Affiliations:** 1 College of the Environment and Ecology, Xiamen University, Xiamen 361102, China; chuyingguo@xmu.edu.cn; 2 Key Laboratory of Ecosystem Network Observation and Modelling, Institute of Geographic Sciences and Natural Resources Research, Chinese Academy of Sciences, Beijing 100101, China; huangrong23@mails.ucas.ac.cn; 3 College of Resources and Environment, University of Chinese Academy of Sciences, Beijing 100190, China; 4 Department of Global Development, College of Agriculture and Life Sciences, Cornell University, Ithaca, NY 14853, USA; peiyucao@cornell.edu; 5 Department of Global Development, Cornell Atkinson Center for Sustainability, Cornell University, Ithaca, NY 14853, USA; 6 College of Life Sciences, University of Chinese Academy of Sciences, Beijing 100049, China; luowenxing20@mails.ucas.ac.cn

**Keywords:** plant phenology, vegetation index, autumn phenology, photosynthetic capacity, vegetation composition, canopy structure

## Abstract

Different approaches have been developed to assess the phenological dynamics of ecosystems. However, diverse data sources and extraction methods for assessing ecosystem phenology can result in discrepant and inaccurate results, especially across different types of vegetation under various climate classifications. Based on the phenology of dominant plant species (Phe_plant_) obtained from ground monitoring in an alpine shrub meadow at Haibei Station (HBS) on the Qinghai–Tibetan Plateau and in a broad-leaved Korean pine forest at Changbai Mountain (CBF) in Northeastern China, we extracted vegetation phenology from the Normalized Difference Vegetation Index (Phe_NDVI_) and photosynthetic phenology from gross primary productivity (Phe_GPP_) using five common methods. These methods included Gaussian fitting, single logistic function fitting, double logistic function fitting, and smoothing techniques combined with fixed threshold and derivative-based determination approaches. There was no consistent interannual trend in either plant phenology or environmental factors at the two sites. Among the three types of plant phenology, a similar interannual pattern in the start of the growing season (SOS) was observed, whereas the interannual patterns for the end of the growing season (EOS) and the growing season length (GSL) were asynchronous. Compared to Phe_plant_, both Phe_NDVI_ and Phe_GPP_ exhibited an earlier SOS, a delayed EOS, and consequently an extended GSL. The SOS derived from both Phe_NDVI_ and Phe_GPP_ was advanced by increasing spring temperatures at both sites, while the relationship between EOS and air temperature was relatively weak. The discrepancy between Phe_NDVI_ and Phe_GPP_ was more pronounced at CBF than at HBS, likely due to the complex vegetation composition and structure of the mixed forest. The different extraction methods produced more consistent and less variable estimates of SOS compared to EOS and GSL at both sites. Among the five methods, the dynamic threshold approach showed a relatively small difference between Phe_NDVI_ and Phe_GPP_, suggesting that it could provide a more consistent estimate of plant phenology across the two sites. This study clearly reveals the inherent discrepancies associated with using different types of phenological data and the influence of extraction methods on phenology across different plant functional types. More attention should be given to improving the accuracy of EOS and understanding the influence of vegetation composition on phenological variation in future studies.

## 1. Introduction

Vegetation phenology is a key biological indicator for understanding ecosystem processes and functions in response to environmental change [1,2,3,4,5]. Phenology not only impacts ecosystem productivity by determining the start, end and length of vegetation growth [6,7,8], but also reflects the adaptive capacity of plants by balancing carbon uptake against environmental stress [1,5,9]. Therefore, phenological variation and its relationship with ecosystem carbon uptake and productivity have received increasing attention in the context of climate change [2,7,10,11,12].

To quantify phenological variation and to support its prediction, several methods have been developed from the plot scale to the global scale over the past few decades based on different observation approaches. Currently, phenological studies primarily utilize three categories of data sources: (1) ground-based observations of species-specific or dominant plant phenology, derived from traditional visual examination [13,14]; (2) flux-based photosynthetic phenology, extracted from gross primary productivity (GPP) using continuous ecosystem flux measurements [15,16,17,18]; and (3) remotely sensed vegetation growth phenology, which encompasses vegetation indices derived from satellite imagery [19,20] or data from digital cameras [21,22,23,24,25,26]; of these, the Normalized Difference Vegetation Index (NDVI) has been the most widely used satellite data source [2,4,20,27,28,29,30,31,32].

Various methods are used to extract vegetation phenology from flux data or satellite imagery [33,34,35]. The process generally involves two basic steps. The first is to fit the time series data through smoothing, which includes function-based methods such as logistic functions [32] and Gaussian functions [7,36], as well as filter-based methods such as the Savitzky–Golay filter [37] and changing-weight filter [33]. The second step is to derive phenological indicators using methods such as the threshold method [38], curvature method [32] or moving average method [39]. Most current studies use a single method or a combination of different methods to extract phenological indicators [35].

The use of diverse data sources, smoothing algorithms, and extraction criteria has led to significant uncertainties in derived phenological metrics, a challenge that has garnered increasing attention [2,19,34,40,41,42]. Several studies have indicated that phenological indicators derived from remote sensing were inconsistent with those derived from flux data [43,44,45,46]. Even when using only satellite data from different sensors, inconsistent and even opposite patterns in derived phenology have been observed due to differences in spatial scale, temporal resolution, and sensor sensitivity to atmospheric conditions [2,19]. These uncertainties are compounded when different smoothing and extraction methods are applied [2,35,40,41,47,48]. This methodology-driven uncertainty is a global phenomenon. For instance, while North American studies report SOS variations of several months, depending on the extraction algorithm [48], European analyses highlight inconsistencies between remote sensing products (e.g., Sentinel-2) and surface-based data (e.g., flux towers and ground networks) during phenological metric calibration [34]. Given these evident inconsistencies [2,4,19,35,42,43], integrated comparisons across data sources and extraction methods in diverse vegetation types are crucial to clarify the relationships among derived phenological metrics [19,40,43].

The influence of data and method choices is evident in reported trends. For example, satellite-based analyses in temperate China indicate trends toward an earlier start of season (SOS) and a delayed end of season (EOS) [49]. However, the magnitude of these shifts is highly dependent on the data sources and extraction methods employed. In European temperate forests, comparisons of remote sensing products with ground records reveal that while the direction of change is consistent, satellite-derived data systematically underestimate the actual dates of phenological events [50,51]. Similarly, North American studies demonstrate that different extraction algorithms produce widely divergent phenological metrics, with the resulting bias varying substantially across ecosystem types [48].

Previous studies indicated that, with global warming, the start of the growing season (SOS) has advanced in both the Tibetan Plateau [52,53] and Northeastern China [45,54,55,56], and the EOS in temperate China has been delayed [57]. Despite the general warming trend, some studies have argued that there has been no detectable trend in phenological variation over the past two decades in the Tibetan Plateau [58,59], with some research even indicating that warming can result in a delayed spring phenology [29]. These conflicting findings underscore the critical influence of data sources and extraction methodologies [1]. These methodology-driven uncertainties represent a broader global pattern, as exemplified by high-latitude Northern Hemisphere growing season assessments, which reveal markedly different trends in prolongation depending on the indicator used (e.g., thermal conditions versus photosynthetic activity) [60]. At the same time, large spatial variations in plant phenology have also been demonstrated due to the influence of land use and vegetation type heterogeneity in the Tibetan Plateau and in temperate China [47,55,61,62,63,64].

To objectively evaluate the relative effects of data source selection versus extraction method choice on derived phenology, and to assess whether these effects are consistent across distinct ecosystems, this study selected two contrasting sites: a temperate broad-leaved Korean pine forest in Northeastern China and an alpine shrubland meadow on the Qinghai–Tibetan Plateau. Using long-term (since 2003) ecosystem carbon flux measurements, satellite NDVI data, and ground phenology records, we applied five widely used phenology extraction methods—Gaussian smooth-threshold detection (GST), double logistic smooth-threshold detection (DLT), double logistic smooth with third-order derivative of curvature (DTC), single logistic smooth with third-order derivative of curvature (STC), and single logistic smooth with second-order derivative of curvature (SSC). Our objectives are to (1) characterize interannual phenological variation at these sites; (2) quantify the agreement among phenological indicators derived from NDVI, CO_2_ flux, and ground records; and (3) assess the relative sensitivity of phenology extraction to different data fitting and determination methods.

## 2. Results

### 2.1. Variations in Environmental Factors and Phenological Indicators

**Figure 1:**
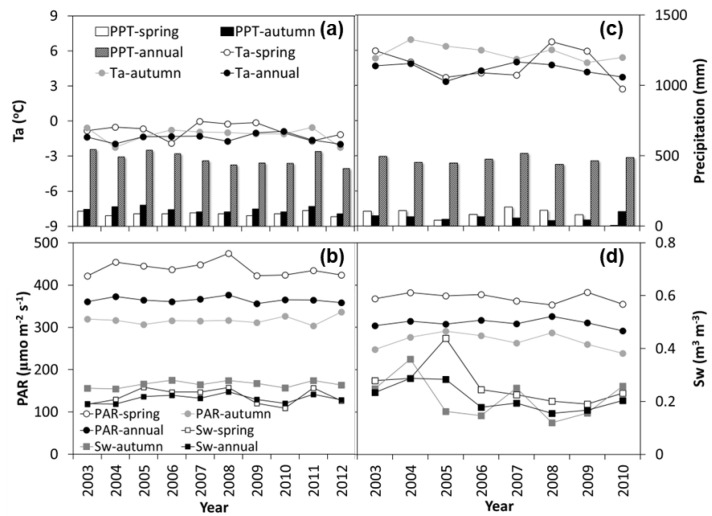
Interannual variations in air temperature (Ta), annual precipitation (PPT), photosynthetically active radiation (PAR) and soil moisture (Sw) across spring, autumn and the entire year at the two study sites. (**a**,**b**) Haibei alpine shrubland (HBS; 2003–2012); (**c**,**d**) Changbai Mountain temperate mixed forest (CBF; 2003–2010).

The interannual variations in environmental factors, including air temperature (Ta), annual precipitation (PPT), photosynthetically active radiation (PAR), and soil moisture (Sw), are presented in Figure 1. To explore variations during key phenological phases, we also present values for each factor aggregated for spring, autumn, and the entire year. Although apparent interannual fluctuations were observed during different seasons, no consistent temporal trend was found for any of the environmental factors.

### 2.2. Comparison of Phenological Variations Derived from GPP, NDVI and Ground Record Data

**Figure 2:**
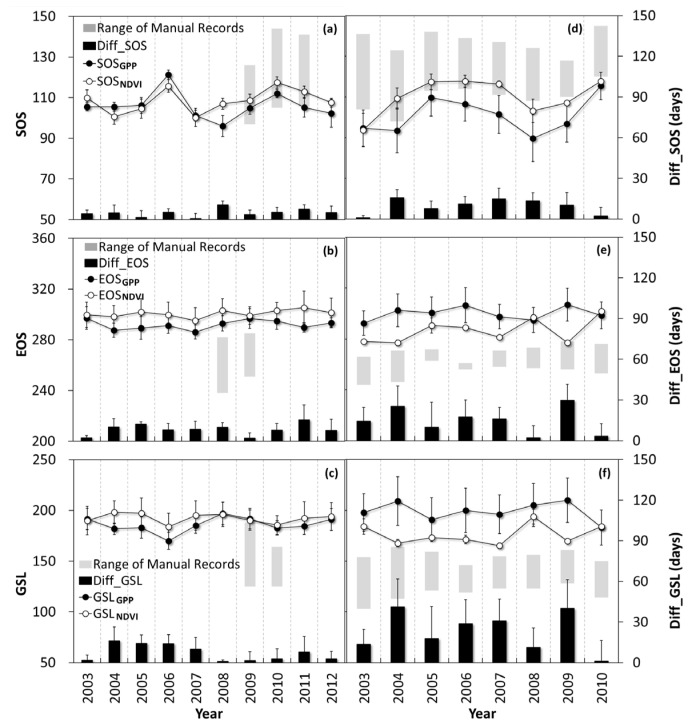
Variations in phenological indicators (SOS, EOS and GSL) derived from GPP (Phe_GPP_) and NDVI (Phe_NDVI_) in an alpine shrubland (HBS) and a temperate mixed forest (CBF). (**a**–**c**) SOS, EOS and GSL at HBS, respectively; (**d**–**f**) SOS, EOS and GSL at CBF, respectively. Filled circles and open circles represent the averages of Phe_GPP_ and Phe_NDVI_, respectively, from the different extraction methods listed in Table 3. The light grey squares indicate the range of phenological indicators for the dominant species at each site, derived from ground-based manual records of plant phenology. The dark grey histograms show the differences between the averages of Phe_GPP_ and Phe_NDVI_. Error bars indicate ±one standard deviation.

The variations in phenological indicators (SOS, EOS and GSL) derived from GPP, NDVI, and ground observations are presented in Figure 2. Overall, the phenological transit dates showed no consistent advancing or delaying trends in Phe_plant_, Phe_GPP_ or Phe_NDVI_ at either site during the study period. Moreover, the patterns of variation differed between the two ecosystems. At HBS, except for a slight delay in SOS_GPP_ relative to SOS_NDVI_ during 2004–2006, both SOS_GPP_ and EOS_GPP_ generally occurred earlier than SOS_NDVI_ and EOS_NDVI_, resulting in only small differences between GSL_GPP_ and GSL_NDVI_. At CBF, SOS_GPP_ consistently occurred earlier than SOS_NDVI_, whereas EOS_GPP_ lagged behind EOS_NDVI_, leading to a longer GSL_GPP_ compared to GSL_NDVI_.

Compared to the ground phenological records of dominant species (Figure 2), both SOS_GPP_ and SOS_NDVI_ tended to align more closely with the earlier sprouting dates at both sites, while both EOS_GPP_ and EOS_NDVI_ were delayed relative to the senescence dates of dominant species. Consequently, both GSL_GPP_ and GSL_NDVI_ were longer than the growth periods of the dominant species. At the same time, Phe_NDVI_ was much closer to the ground records than Phe_GPP_ at CBF (Figure 2d–f). Compared to the available complete ground records from 2009 at HBS, the average SOS_GPP_ and SOS_NDVI_ were advanced by 5 and 1 days, respectively, while the average EOS_GPP_ and EOS_NDVI_ were delayed by 33 and 35 days, resulting in extensions of GSL_GPP_ and GSL_NDVI_ by 38 and 36 days, respectively. Compared to the ground records at CBF, the average SOS_GPP_ and SOS_NDVI_ were advanced by 22 and 12 days, respectively, while the average EOS_GPP_ and EOS_NDVI_ were delayed by 36 and 20 days, also resulting in extensions of GSL_GPP_ and GSL_NDVI_ by 58 and 32 days, respectively.

In general, the start dates of both photosynthetic and vegetation phenology were earlier, and the end dates later, than those of the dominant species phenology. The consistency between photosynthetic phenology and vegetation phenology was more pronounced at HBS than at CBF (Figure 2). For example, the multi-year average differences in SOS, EOS and GSL between Phe_GPP_ and Phe_NDVI_ at HBS were approximately 2, 9 and 6 days, respectively, whereas these differences were larger at CBF (Figure 2d–f), reaching about 9, 13 and 23 days, respectively (Table 1).

**Table 1:** Comparisons of growing season indicators (SOS, EOS, GSL) derived from the vegetation index (Phe_NDVI_) and photosynthetic activity (Phe_GPP_). All values, including the multi-year averages for each method and the “Phe_GPP_-Phe_NDVI_” differences, are represented as mean ± one standard error. Superscript letters indicate significant differences (*p* < 0.05) among methods within the same data source and indicator, while an asterisk (*) denotes a significant difference between Phe_GPP_ and Phe_NDVI_ for a given indicator and method.

Site	Indicator	Data Source	DLT	GST	DTC	STC	SSC	Mean	Phe_GPP_-Phe_NDVI_
HBS	SOS	GPP	109 ± 7 ^a^	107 ± 6 ^a^	107 ± 8 ^a^	103 ± 8 ^a^	103 ± 8 ^a^	106 ± 7	−2 ± 0.6
		NDVI	112 ± 6 ^ab^	112 ± 5 ^ab,^*	106 ± 6 ^b^	106 ± 6 ^b^	106 ± 6 ^b^	108 ± 6	
	EOS	GPP	284 ± 4 ^a^	288 ± 3 ^a^	293 ± 5 ^b^	297 ± 5 ^b^	297 ± 5 ^b^	292 ± 7	−9 ± 0.7
		NDVI	290 ± 3 ^b,^*	290 ± 3 ^b^	307 ± 5 ^a,^*	308 ± 3 ^a,^*	308 ± 3 ^a,^*	301 ± 9 *	
	GSL	GPP	174 ± 8 ^a^	181 ± 6 ^ab^	186 ± 10 ^b^	194 ± 9 ^b^	194 ± 9 ^b^	186 ± 11	−6 ± 0.9
		NDVI	178 ± 5 ^b^	178 ± 4 ^b^	201 ± 6 ^a,^*	202 ± 5 ^a,^*	202 ± 6 ^a,^*	192 ± 13 *	
CBF	SOS	GPP	113 ± 7 ^a^	108 ± 8 ^a^	94 ± 9 ^a^	95 ± 10 ^a^	94 ± 10 ^a^	101 ± 12	−9 ± 0.8
		NDVI	114 ± 7 ^a^	114 ± 7 ^a^	108 ± 10 ^a,^*	108 ± 10 ^a,^*	108 ± 10 ^a,^*	110 ± 9 *	
	EOS	GPP	285 ± 3 ^a^	290 ± 4 ^a^	311 ± 6 ^b,^*	306 ± 7 ^b,^*	306 ± 7 ^b,^*	300 ± 12 *	13 ± 1.6
		NDVI	286 ± 9 ^ab^	287 ± 10 ^a^	286 ± 10 ^ab^	287 ± 10 ^a^	287 ± 10 ^a^	286 ± 9	
	GSL	GPP	172 ± 7 ^a^	182 ± 8 ^a^	218 ± 9 ^b,^*	212 ± 11 ^b,^*	212 ± 11 ^b,^*	199 ± 21 *	23 ± 2.1
		NDVI	172 ± 8 ^ab^	173 ± 9 ^ab^	178 ± 11 ^a^	179 ± 12 ^a^	179 ± 12 ^a^	176 ± 10	

### 2.3. Phenological Indicators Derived from Different Extraction Methods

The phenological indicators from different data fitting and determination methods are also presented in Table 1. Apparent differences were observed between the threshold and curvature approaches. Compared to the indicators determined by DTC, STC and SSC, the SOS derived from both GPP and NDVI by the threshold methods (DLT and GST)—except for NDVI-derived indicators at CBF—was delayed, while the EOS from the threshold methods was significantly earlier, resulting in a significantly shorter GSL.

The variability of different extraction methods for each data source during the study period was also examined. At HBS, the average maximum deviations in SOS, EOS and GSL were 8 ± 3, 14 ± 3 and 20 ± 3 days for GPP-derived data, and 7 ± 2, 19 ± 3 and 25 ± 4 days for NDVI-derived data, respectively. At CBF, the average maximum deviations in SOS, EOS and GSL were 20 ± 3, 26 ± 3 and 46 ± 4 days for GPP-derived data and 7 ± 5, 5 ± 3 and 9 ± 5 days for NDVI-derived data, respectively.

Regarding the differences between Phe_GPP_ and Phe_NDVI_ derived from different methods, most differences were insignificant for both DLT and GST. However, using DTC, STC and SSC, EOS_NDVI_ was significantly delayed compared to EOS_GPP,_ leading to a significant increase in GSL_NDVI_ relative to GSL_GPP_ at HBS. In contrast, at CBF, a significantly extended GSL_GPP_ was observed, resulting from the earlier SOS_GPP_ and significantly later EOS_GPP_ compared to the SOS and EOS derived from NDVI (Table 1).

### 2.4. Environmental Controls on the Variation in Phenology

**Figure 3:**
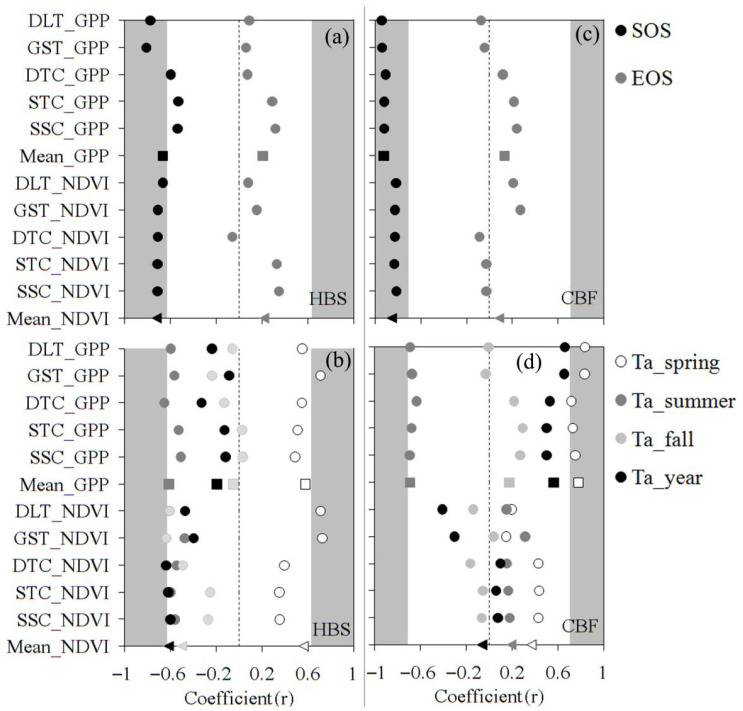
Correlation coefficients (r) of air temperature with phenological transition dates at HBS (**a**,**b**) and CBF (**c**,**d**). Panels (**a**,**c**) show correlations for SOS and EOS, derived from their relationships with mean air temperature in spring and fall, respectively. Panels (**b**,**d**) show correlations for GSL, derived from its relationship with mean air temperature in spring (Ta_spring), summer (Ta_summer), fall (Ta_fall), and the entire year (Ta_year). The grey area denotes non-significant relationships (*p* > 0.05). Squares indicate coefficients derived from the mean phenological dates based on GPP (Mean_GPP); triangles indicate coefficients from the mean phenological dates based on NDVI (Mean_NDVI), corresponding to the methods listed in Table 3.

The influence of environmental factors on phenological indicators is shown in Figure 3. Spring air temperature (Ta_spring) had a significant effect on the SOS of both Phe_GPP_ and Phe_NDVI_ at the two sites (Figure 3a,c), except for the SOS of Phe_GPP_ derived using DTC, STC and SSC at HBS. However, the relationship between the EOS—whether from Phe_GPP_ or Phe_NDVI_—and fall air temperature (Ta_fall) was very weak. No significant relationships were observed between GSL and summer air temperature (Ta_summer), Ta_fall, or annual air temperature (Ta_year). Nevertheless, a positive effect of Ta_spring on GSL was observed for the GSL derived using the threshold method at HBS and for the GSL derived using GPP at CBF.

### 2.5. Influences of Data Sources and Extraction Methods on Phenological Indicators

**Figure 4:**
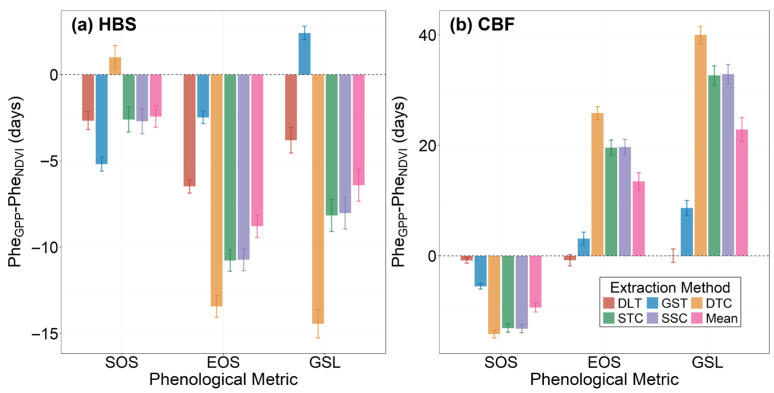
Differences between Phe_GPP_ and Phe_NDVI_ from different extraction methods (see Table 3) for the (**a**) HBS and (**b**) CBF sites across three phenological metrics (SOS, EOS, GSL). Error bars indicate ±one standard error.

The phenological indicators derived from GPP and NDVI were significantly affected by the extraction methods, as presented in Table 3 and Figure 4. Figure 4 presents the difference between the Phe_GPP_ and Phe_NDVI_ values derived from the five methods. It was clear that the delta (Phe_GPP_ minus Phe_NDVI_) from both DLT and GST, which employ the dynamic threshold detection method, was smaller than that from DTC, STC and SSC, especially for EOS and the subsequent GSL.

**Figure 5:**
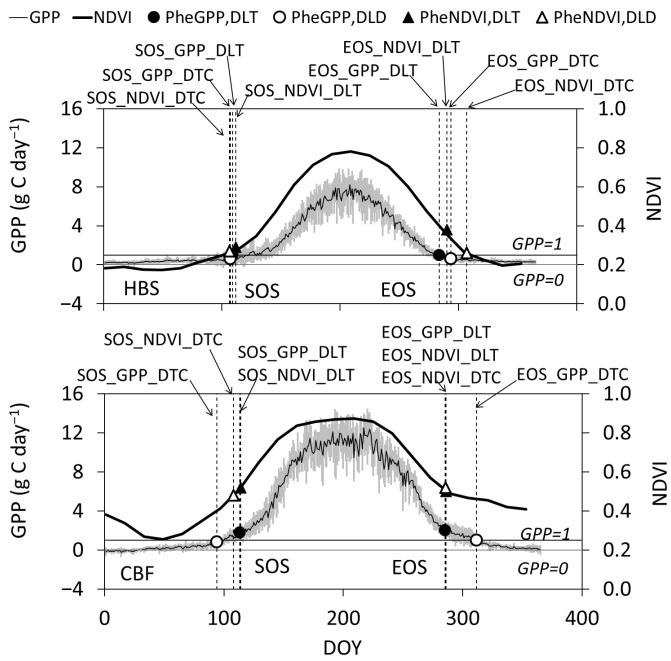
Comparison of phenological transition dates determined by the DLT (dynamic threshold) and DTC (curvature) methods using GPP and NDVI data at HBS (**upper panels**) and CBF (**lower panels**). Bold and thin lines represent seasonal NDVI and GPP, respectively. Filled circles and triangles denote SOS/EOS determined by DLT from GPP (Phe_GPP, DLT_) and NDVI (Phe_NDVI, DLT_), respectively. Open circles and triangles denote SOS/EOS determined by DTC from GPP (Phe_GPP, DTC_) and NDVI (Phe_NDVI, DTC_), respectively. The grey area indicates ±one standard deviation of GPP during the study period.

Using the average daily GPP and NDVI during the study period, Figure 5 presents a comparison of the phenological indicators determined by DLT and DTC, which represented the threshold and curvature method, respectively. At HBS, the SOS_GPP_ and SOS_NDVI_ results extracted from either DLT or DTC were close. However, there were apparent differences for EOS, especially from DTC. At CBF, apparent differences between SOS_GPP_ and SOS_NDVI_ were observed, with the opposite pattern occurring in autumn. A comparison between the phenological dates derived from GPP and the fixed threshold (GPP = 1 gC m^−2^ d^−1^) revealed (Figure 5): At HBS, the SOS and EOS derived from GPP closely aligned with the threshold dates, whereas the EOS derived from NDVI significantly lagged behind the date when GPP declined to this threshold. At CBF, only the SOS and EOS derived from GPP using the DTC method coincided with the threshold dates, while deviations were observed in the results extracted by the DLT method.

### 2.6. Convergence of Phenological Indicators Derived from Different Data Sources and Methods

**Figure 6:**
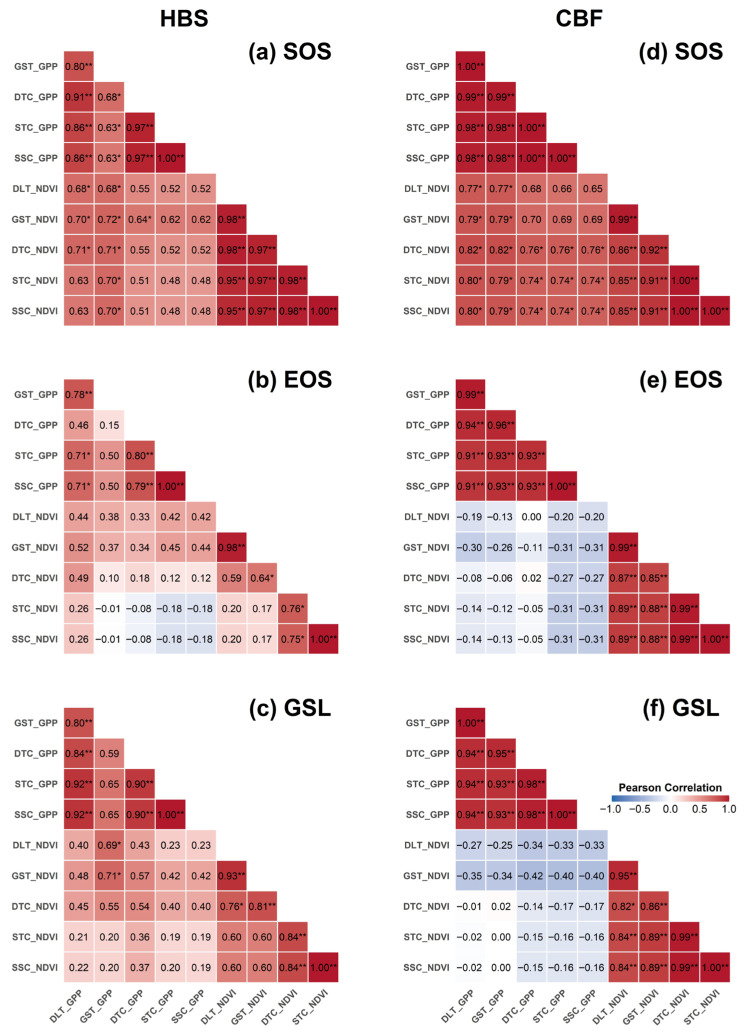
Correlation analysis of phenological indicators across different extraction methods. Panels (**a**–**c**) represent SOS, EOS, and GSL at HBS (**left**), while panels (**d**–**f**) represent SOS, EOS, and GSL at CBF (**right**), respectively. Each panel is divided by two orthogonal white lines into three sections: the lower left quadrant displays correlations between indicators derived from GPP and NDVI; the upper left quadrant shows correlations among indicators derived exclusively from GPP; and the lower right quadrant shows correlations among indicators derived exclusively from NDVI. Asterisks indicate statistical significance as follows: * for *p* < 0.05, ** for *p* < 0.01, and no symbol for non-significance.

Figure 6 further shows the convergence among the indicators from GPP and NDVI with different extraction methods using regression analysis. First, a general positive correlation was observed for the SOS or EOS derived from the same data source at both sites. However, this correlation for GSL tended to be less evident due to the combined effect of SOS and EOS determination. Second, a significant positive correlation was usually valid only for the SOS derived by different methods using GPP and NDVI, and no relation or even a negative relationship was exhibited for EOS and GSL, especially at CBF, which has a more complex community structure than HBS.

## 3. Discussion

### 3.1. Environmental Controls on the Variation in Plant Phenology

Temperature is widely recognized as a determining factor of phenological variations from the leaf to the global scale [4,27,56]. Previous studies have indicated that phenological variations, particularly advanced SOS, are primarily associated with rising temperatures on the Tibetan Plateau and in the temperate regions of China [1,14,54], while precipitation also plays a non-negligible role in arid areas [47,57,62,64,65]. Consistent with these findings, our study identified spring temperature as the key environmental factor controlling SOS at both sites.

However, our research also revealed a key complexity: the decoupling of EOS from fall temperatures. The weak relationship observed between EOS and fall air temperature in this study highlights the ongoing challenge in accurately determining EOS [1,3,4,66]. Since GSL depends on both SOS and EOS, further research is needed to improve the accuracy of EOS estimation and enhance our understanding of how environmental conditions influence EOS variations [57,67,68]. For example, incorporating the redness index could significantly improve EOS estimation when using digital cameras [69]. Meanwhile, data presented in Figure 3, such as air temperature across different seasons, also show that divergent results can arise depending on the methods used to extract phenological transition dates and the environmental factors considered [47]. This underscores the importance of fully accounting for methodological and factor selection when interpreting and comparing phenological studies. Furthermore, the integrated phenological signal (Phe_GPP_/Phe_NDVI_) represents the collective response of the plant community. Species-specific sensitivities, such as the strong temperature dependence of leaf-out in deciduous trees like *Quercus mongolica* at CBF, or the potential influence of moisture availability on the growth initiation of alpine meadow species like *Kobresia* spp. at HBS, are averaged within these canopy-level metrics. This species-aggregation effect may partially explain the sometimes weak or divergent relationships between environmental factors (like fall temperature) and ecosystem-level EOS.

### 3.2. Phenological Patterns from Different Data Sources

Based on the phenological indicators derived from continuous GPP and NDVI measurements and ground records, no evident phenological shifts were observed at HBS and CBF (Figure 2). The absence of clear phenological trends in this study may be attributed to two main reasons. First, the interannual variation in the main environmental factors did not exhibit a continuous increasing or decreasing trend within different seasons during the study period (Figure 1). Second, the trends of phenological shifts are closely related to the time frame analysed, which has been observed in the Northern Hemisphere [1,70], the temperate region of China [28] and the Tibetan Plateau [29,52]; notably, a decelerating or reversing pattern of the spring phenological shift during the warming hiatus that began in the 2000s has been recently detected [71], which generally coincides with the periods in this study. Therefore, such discrepancies suggest potential differences in phenological patterns across different spatial scales and temporal periods [40].

The differences in the magnitudes of phenological indicators derived from the records of NDVI, GPP and ground observations were mainly related to the different implications of the derived phenological indicators. Compared to the green-up of dominant species, an advanced SOS and a delayed EOS were derived from both GPP and NDVI. Previous studies also found that SOS was approximately 20 days earlier when using MODIS NDVI compared to ground records [27]. This finding is consistent with the influence of nondominant species, especially in the understory, which can green up earlier than the dominant canopy [19,70]. The early sprouting of nondominant understory species can trigger the initial greenness of vegetation and ecosystem photosynthetic processes, which can be detected by remote sensing and flux measurements, respectively. Meanwhile, the late senescence of nondominant species can maintain a certain amount of plant leaves and photosynthetic activity temporarily. Additionally, such phenomena potentially reflect the temporal strategies of ecological niches from different species, and the dominant species generally use the favourable period to avoid adverse conditions that frequently occur in early spring and late autumn in the community.

Regarding the apparent difference between Phe_GPP_ and Phe_NDVI_ at CBF, the advancement of SOS_GPP_ and delay of EOS_GPP_ compared to SOS_NDVI_ and EOS_NDVI_ were probably related to the specific forest community composition, particularly the contribution of the cold-tolerant evergreen conifer *Pinus koraiensis* Siebold & Zucc. (Korean pine). The considerable photosynthetic capacity of evergreen coniferous species can not only be sustained even under low temperatures [72,73,74] but also recover quickly with increasing temperature in early spring. At the same time, the green-up of the understory could be missed by satellite sensing due to the cover of evergreen Korean pine, contributing to the advanced photosynthesis [22]. This situation was different from that of the deciduous forest, in which the defoliation time threshold detection strongly depended on the spatial and temporal distribution of understory plants [66], and the green-up of the understory could be monitored several weeks before bud burst of the trees [13,70]. However, most herbaceous species at HBS had already finished their life history, which caused a quick reduction in the leaf photosynthetic capacity even before substantial leaf drop was detected by remote sensing. Therefore, the differences between Phe_GPP_ and Phe_NDVI_ were probably related to the specific community composition at HBS. This includes the presence of the dominant shrub *Potentilla fruticosa*, which maintains photosynthetic capacity late into the season, contrasted with the phenology of co-dominant herbaceous species such as *Kobresia* spp. These contrasting life forms (deciduous shrubs vs. perennial sedges) contribute differently to the integrated canopy signal captured by GPP and NDVI.

### 3.3. Effects of Data Sources and Extraction Methods on Phenological Indicators

Our results demonstrate that the five extraction methods (GST, DLT, DTC, STC, SSC) produced notably different estimates of SOS, EOS, and GSL, with the most pronounced variations observed in EOS and GSL (Table 1; Figure 4 and Figure 5). As shown in Table 3, the phenological indicators derived from GPP and NDVI were significantly influenced by the extraction methods. Similar to previous studies conducted using satellite vegetation index data [41,47,48,75], this study revealed apparent differences among various data fitting and determination methods based on NDVI. This divergence stems directly from their underlying mathematical principles. The discrepancies observed in Figure 4 and Figure 5 are closely associated with the criteria of different methods and seasonal patterns of GPP or NDVI. The threshold method considers the phenological transit date to be the time when GPP or NDVI reaches a certain proportion of the amplitude [29,38]. Threshold-based methods (DLT, GST) generally yielded later SOS, earlier EOS, and consequently a shorter GSL compared to curvature-based methods (DTC, STC, SSC). In contrast, the derivative method examines the changing rate of GPP or NDVI during spring and autumn [53,58], making it more sensitive to environmental variations but also introducing more uncertainty in determining phenological transit dates [25]. Additionally, due to the small differences within threshold methods or curvature methods, discrepancies in derived phenology potentially resulted from the data source and detection method [76], while the contribution from data smoothing was limited. Notably, the dynamic threshold methods (DLT and GST) showed smaller discrepancies between Phe_GPP_ and Phe_NDVI_, suggesting their potential for cross-data harmonization in multi-source phenology studies. Therefore, in this study, the dynamic threshold methods yielded smaller discrepancies between Phe_NDVI_ and Phe_GPP_, suggesting their potential for providing more consistent cross-data estimates of plant phenology. At the same time, it should be noted that this study emphasized the comparison of plant phenology derived from diverse data sources and extraction methods; threshold optimization through more complicated approaches was not included. Instead, a common standard based on a review of previous studies was adopted, with thresholds set at 10% of the annual amplitude for GPP and 20% for NDVI.

The distinct patterns observed at HBS and CBF can be interpreted in light of their ecosystem characteristics. At HBS, the close agreement between SOS_GPP_ and SOS_NDVI_ can be attributed to the rapid green-up and recovery of the photosynthetic capacity, which caused both GPP and NDVI to increase synchronously. The apparent differences in EOS, especially from DTC, are probably related to the reduction in photosynthetic capacity even before leaf colouring in autumn due to the decline in temperature or radiation [17,77]. At CBF, the earlier SOS_GPP_ derived by DTC can be attributed to the recovery of photosynthesis in the evergreen *Pinus koraiensis* and the green-up of understory vegetation, which occurs before the bud burst of the dominant deciduous canopy species such as *Quercus mongolica* is fully detectable by NDVI. The opposite pattern observed in autumn further underscores the complex phenological dynamics in forests with multi-layered canopies. Thus, the phenological indicators derived by these two types of methods are significantly different, especially in ecosystems with complex community compositions, such as forests.

For the phenology derivation from flux measurements, GPP = 1 gC m^−2^ d^−1^ was assumed to be the start or end of the growing season [17]. Regarding the applicability of this fixed threshold approach, this study found that at CBF, deviations occurred between the SOS or EOS derived from GPP using the DLT method and the fixed threshold of GPP = 1 gC m^−2^ d^−1^. The discrepancy was probably related to the larger amplitude of GPP at CBF than that at HBS, which resulted in more time to attain the detectable threshold. Therefore, this phenomenon potentially implies that the applicability of the constant threshold, such as GPP attaining 1 gC m^−2^ d^−1^, in the determination of phenology across different biomes still needs further consideration. To compare the widely used methods, we directly adopted fixed thresholds of 10% and 20% from previous studies [15,35] to determine phenology from GPP and NDVI. However, given that climatic ranges are shifting under climate change [78], these fixed thresholds may no longer accurately capture actual phenological events. Therefore, more robust statistical approaches, such as the Akaike Information Criterion (AIC) and the Bayesian Information Criterion (BIC), could be employed to derive more reliable estimates of phenological variations.

### 3.4. Differences of Phenological Indicators Derived from Diverse Data Sources and Methods

Beyond the choice of extraction method, the fundamental relationship between the two major data sources (GPP and NDVI) is of great interest. Theoretically, there should be a close relationship between phenological indicators derived using different methods from GPP and NDVI. Yet, our findings reveal systematic discrepancies: interannual trends (Figure 2) and magnitudes (Table 1) indicated varying degrees of differences in phenological indicators (Figure 4). This observed inconsistency between data sources underscores a critical challenge. Given the wide use of continuous GPP and NDVI data in the analysis of phenology shifts, how to effectively harmonize these two types of data needs to be further studied [40,43].

## 4. Data Sources and Methods

### 4.1. Study Sites

**Figure 7:**
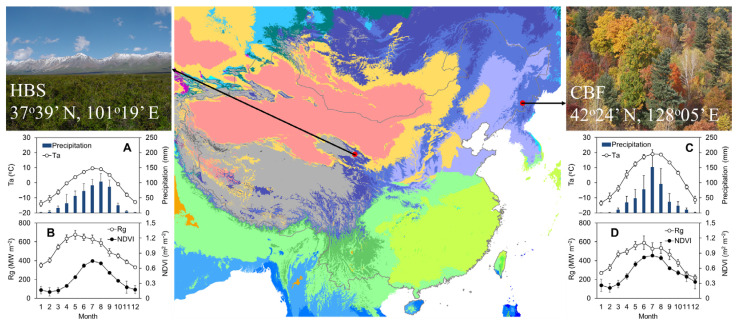
Location, climate and seasonal dynamics of the study sites. (**A**,**B**) Alpine shrubland meadow at Haibei (HBS) on the Qinghai–Tibetan Plateau. (**C**,**D**) Broad-leaved Korean pine forest at Changbai Mountain (CBF) in Northeastern China. Panels (**A**,**C**) show seasonal variations in air temperature (Ta, open circles) and precipitation (blue columns). Panels (**B**,**D**) show seasonal variations in gross solar radiation (Rg, open circles) and the Normalized Difference Vegetation Index (NDVI, black circles). Error bars represent ±one standard deviation. The background map depicts the Köppen–Geiger climate classification from Beck et al. (2023) [79]. Site photographs are shown in the inset panels.

In this study, a temperate broad-leaved Korean pine forest at Changbai Mountain (CBF) in Northeast China and an alpine shrubland at Haibei station (HBS) on the Qinghai–Tibetan Plateau were selected as research sites (Figure 7 and Table 2). According to the Köppen–Geiger climate classification proposed by Beck et al. (2023) [79], the climates at CBF and HBS were classified as Dwb (cold, dry winter, warm summer) and Dwc (cold, dry winter, cold summer), respectively.

CBF is one of the most important forest areas, covering approximately 50,000 hectares in this region. Under the influence of the temperate continental monsoon climate in East Asia, both precipitation and temperature in CBF reach their highest levels in summer (June to August), showing a clear synchronous pattern in water and heat conditions (Figure 7C,D), which also influences canopy development (Figure 7D). According to the meteorological data from 1985 to 2005, the mean annual temperature is 3.6 °C, with a mean of 20.5 °C in August and −16.5 °C in January (Figure 7C). As a mature forest (forest age > 150 years) that is considered the zonal vegetation in this region, CBF maintains a strong carbon sequestration capacity based on flux measurements [80,81,82]. HBS is mainly influenced by the highland continental climate. The solar radiation is intense, with an annual total reaching 6139.2 MJ/m^2^, which is considerably higher than the 5093 MJ/m^2^ at CBF. HBS also shows a clearly synchronous variation in water and heat conditions (Figure 7A,B). Based on meteorological data from 1980 to 2010, precipitation mainly occurs from May to October, and the mean annual temperature is −1.0 °C, with a mean of 10.4 °C in July and −14.4 °C in January (Figure 7A). As one of the representative vegetation types dominated by *Potentilla fruticosa* L. in this region, long-term eddy flux measurements indicated that HBS acts as a weak carbon sink, with large interannual variability in fluxes due to environmental changes [81,83,84].

**Table 2:** Summary of site descriptions for the alpine shrubland meadow (HBS) and the broad-leaved Korean pine forest (CBF).

Site	HBS ^1^	CBF ^2^
Location	37°39′ N, 101°19′ E	42°24′ N, 128°05′ E
Elevation (m a.s.l.)	3293	738
Area (hectares)	>100,000	~50,000
Topography	Flat	Flat
Climate classification ^3^	Cold, dry winter, cold summer	Cold, dry winter, warm summer
Mean annual temperature (°C)	−1.0	3.6
Annual precipitation (mm) ^4^	570	695
Ecosystem type	Alpine shrub-meadow	Temperate mixed forest
Dominant species ^5^	*Potentilla fruticosa* L., *Festuca ovina* L., *Potentilla anserina* L., *Carex pachyrrhiza* Franch., *Elymus nutans* Griseb.	*Pinus koraiensis* Siebold & Zucc., *Quercus mongolica* Fisch. ex Ledeb., *Acer mono* Maxim., *Fraxinus mandshurica* Rupr., *Tilia amurensis* Rupr.
Mean canopy height (m)	0.6	26
Maximum leaf area index (m m^−2^)	2.8	6.1
Soil type	Silty clay loam (Mol-Cryic Cambisols)	Montane dark brown forest soil

^1^ Site information for the alpine shrubland at Haibei (HBS) was obtained primarily from Fu et al. (2006) [83] and Li et al. (2016) [84]. ^2^ Site information for the temperate broad-leaved Korean pine forest at Changbai Mountain (CBF) was obtained primarily from Zhang et al. (2006) [82] and Yu et al. (2008) [80]. ^3^ The Köppen–Geiger climate classification is from Beck et al. (2023) [79]. ^4^ Meteorological data for HBS and CBF cover the periods 1980–2010 and 1985–2005, respectively. ^5^ The system of The Plant List (http://www.theplantlist.org/) was utilized for the species nomenclature.

### 4.2. Data Sources

#### 4.2.1. Ecosystem Carbon Flux and Environmental Factors

As pioneering sites of the Chinese flux observation and research network (ChinaFLUX), CBF and HBS have maintained long-term and continuous measurements of ecosystem carbon and water fluxes based on the eddy covariance technique since 2002. The open-path eddy covariance system consisted of a CO_2_/H_2_O infrared analyser (Model LI-7500, LICOR Inc., Lincoln, NE, USA) and a three-dimensional ultrasonic anemometer (Model CSAT3, Campbell Scientific Inc., Logan, UT, USA), both operating at a measurement frequency of 10 Hz. Data were acquired and stored automatically using a data logger (Model CR5000, Campbell Scientific). The flux measurement heights were set at 41.8 m in CBF and 2.0 m in HBS, respectively.

The routine meteorological measurement system included sensors for air temperature and humidity (Model HMP45C, Vaisala, Vantaa, Finland), four-component radiation (Model CNR1, KIPP&Zonen, Delft, The Netherlands), precipitation (Model 52203, RM Young, Traverse City, MI, USA), soil temperature (Model 107, Campbell Scientific) and soil moisture (Model CS616, Campbell Scientific). Soil temperature was measured at depths of 5, 10, 20, 50, and 100 cm at both sites. Soil moisture was measured at depths of 5, 20, and 50 cm in CBF, and at 5, 20, and 40 cm in HBS. This study utilized meteorological and flux data from 2003 to 2010 for CBF and from 2003 to 2012 for HBS.

#### 4.2.2. Normalized Difference Vegetation Index (NDVI)

This study utilized the satellite-derived Normalized Difference Vegetation Index (NDVI) [85,86] for vegetation phenology analysis. NDVI data were downloaded from the Oak Ridge National Laboratory Distributed Active Archive Centre (ORNLDAAC, http://www.daac.ornl.gov) and comprised the MOD13Q1 Vegetation Index products from the Moderate Resolution Imaging Spectroradiometer (MODIS). Each pixel value represented the 16-day maximum NDVI to minimize the influence of cloud cover and atmospheric aerosols. The NDVI dataset was subsequently processed using a noise-filtering method following the approach described in [87].

#### 4.2.3. Ground Record of Plant Phenology

During the research period, the phenology of dominant plants (Phe_plant_) was recorded manually at both sites. Following the protocols of the Chinese Ecosystem Research Network (CERN), the dates for the SOS and EOS of the growing season were defined as when over 10% of individuals for each species exhibited leaf flushing and leaf fading or falling, respectively. We collected the ground records of plant phenology from the data service system of CERN (http://www.cnern.org.cn/data/initDRsearch?classcode=STA) (accessed on 10 March 2024).

The manual phenology records at CBF covered the years from 2003 to 2010, and included seven deciduous tree species: *Quercus mongolica* Fisch. ex Ledeb., *Syringa reticulata var*. *mandshurica* (Maxim.) H.Hara, *Philadelphus schrenkii* Rupr., *Acer mono* Maxim., *Fraxinus mandshurica* Rupr., *Tilia amurensis* Rupr. and *Corylus heterophylla* Fisch. ex Trautv. (Korean pine evergreen trees were excluded). At HBS, records for five species—*Potentilla fruticosa*, *Festuca ovina* L., *Potentilla anserina* L., *Carex pachyrrhiza* Franch. and *Elymus nutans* Griseb.—were available from 2008 to 2011. At this site, the canopy height and shrub coverage were approximately 30–60 cm and 60–80%, respectively, and the height of the secondary layer of grasses was approximately 8–16 cm [84].

### 4.3. Data Processing

#### 4.3.1. Flux Data Processing

The eddy covariance approach is widely accepted as a technique for directly measuring net CO_2_, water vapor and energy fluxes between vegetation and the atmosphere. The net ecosystem exchange (NEE) between vegetation and the atmosphere was calculated using Equation (1), which ignored all advective terms in the mass conservation equation:

(1) $$NEE=\bar{w^{\prime }\rho_{c}^{\prime }(z_{r})}+\int_{0}^{z_{r}}\frac{\partial \bar{\rho_{c}}}{\partial t}dz$$

where the first term on the right-hand side of Equation (1) is the eddy flux of CO_2_, which derived from the covariance between the deviations of both vertical wind velocity (*w*, m s^−1^) and CO_2_ concentration (*ρ*_c_, mmol m^−3^) at the height of flux observation (*z*_r_); the second term is the CO_2_ storage within the air column below *z*_r_ and was only applied to CBF due to the influence of high canopy [80]. The primes and overbar indicate the deviations and the time-averaged mean within each 30 min, respectively [80]. The overbar indicates a time-averaged value.

Uniform data quality control and processing procedures [80] were applied, including planar-fit rotation, ultrasonic virtual temperature correction, and correction for air density fluctuations (WPL correction). To minimize the interference from noise data, we implemented the following screening methods: (1) elimination of flux data during precipitation events; (2) application of a flux data threshold of [−3, 3] to remove obviously abnormal values; (3) exclusion of data under stable conditions using an appropriate u* threshold to reduce effects of insufficient turbulence [88]; and (4) removal of outlier data by comparing differences with the adjacent values [89].

Following quality control and filtering, 73% and 83% of daytime data were valid for HBS and CBF, respectively, while only 24% and 34% of nighttime data were valid for HBS and CBF, respectively. To obtain continuous flux data, we used nonlinear regression to interpolate missing values [90]. Short gaps (<2 h) were filled by linear interpolation. For longer gaps during the daytime growing season, missing data were filled using the Michaelis–Menten equation with a 10-day moving window. Missing nighttime data were filled using the Lloyd & Taylor equation. Detailed procedures are described in [80].

Considering that NEE represents the net carbon sequestration resulting from the gross photosynthetic capacity and total respiration consumption [89], ecosystem GPP is expressed as the sum of NEE and ecosystem respiration (Reco), as shown in Equation (2):

GPP = −NEE + Reco(2)

In flux measurements, a negative NEE indicates carbon sequestration by the ecosystem, while a positive NEE indicates emission. Reco is equivalent to nighttime flux measurements when the ecosystem respires, while daytime ecosystem respiration is estimated through an exponential relationship with temperature [80,89].

#### 4.3.2. Extraction of Phenological Indicators

The SOS, EOS and growing season length (GSL) have received considerable attention due to their close correlation with ecosystem productivity [4]. GPP and NDVI were used to derive SOS and EOS for both HBS and CBF, after which the growing season length (GSL) was defined as the difference between EOS and SOS. The approaches using GPP to derive phenological indicators were referred to as Phe_GPP_, and the GPP-derived SOS, EOS and GSL were referred to as SOS_GPP_, EOS_GPP_, and GSL_GPP_, respectively. Similarly, the approaches that used NDVI were referred to as Phe_NDVI_, and the NDVI-derived SOS, EOS and GSL were referred to as SOS_NDVI_, EOS_NDVI_, and GSL_NDVI_, respectively.

We applied five phenology extraction methods—GST, DLT, DTC, STC, and SSC—to both GPP and NDVI time series. These methods represent common threshold- and curvature-based approaches, allowing a comprehensive comparison of phenological metrics derived from different data sources and algorithms. The specific smoothing functions and threshold/derivative criteria for each method are summarized in Table 3. These methods fall into two broad categories frequently utilized in phenology extraction [38]: the threshold method and the curvature method. Each method combines one of three common data smoothing techniques (double logistic, Gaussian, or single logistic) with a specific rule (threshold or derivative-based) for deriving phenological indicators.

**Table 3:** Methods for deriving phenology indicators.

Method	Abbr.	Algorithm	Reference
Double Logistic smooth-Threshold detection	DLT	Smoothed by Double Logistic, Determined by a certain threshold (Flux-10%, NDVI-20%)	[60,91]
Gaussian smooth-Threshold detection	GST	Smoothed by Gaussian, Determined by a certain threshold (Flux-10%, NDVI-20%)	[92]
Double logistic smooth with Third order derivative of Curvature	DTC	Smoothed by Double Logistic, Determined by the zero point of the third order derivative of curvature in spring and autumn, respectively	[8,15]
Single logistic smooth with Third order derivative of Curvature	STC	Smoothed by Single Logistic, Determined by the zero point of the third order derivative of curvature in spring and autumn, respectively	[32,44]
Single logistic smooth with Second order derivative of Curvature	SSC	Smoothed by Single Logistic, Determined by the zero point of the second order derivative of curvature in spring and autumn, respectively	[61,93]

For the threshold approach using DLT and GST, the SOS or EOS was determined based on a predefined percentage of the annual amplitude (the difference between the annual maximum and minimum values). In this study, we adopted a common standard based on the review of previous studies, setting the threshold at 10% of the annual amplitude for GPP and 20% for NDVI. For the curvature approaches—DTC, STC, and SSC—the SOS and EOS were determined as the Julian day of the zero point in the second- or third-order derivative of the fitted function during spring and autumn, respectively.

### 4.4. Statistics

To compare the phenological indicators derived from different data sources and extraction methods, one-way analysis of variance (ANOVA) was utilized to test the differences, with post hoc comparisons performed using the Least Significant Difference (LSD) test. The Pearson correlation coefficient was applied to analyze the relationship between the extracted phenological indicators and environmental factors.

## 5. Conclusions

Our results show that none of the three phenological types (Phe_plant_, Phe_GPP_, or Phe_NDVI_) exhibited consistent advancing or delaying trends at either site during the study period, likely due to fluctuations in environmental conditions. While spring temperature played a determining role in shaping SOS patterns in both Phe_GPP_ and Phe_NDVI_, no dominant controlling factors were identified for EOS or GSL. Compared to Phe_GPP_ and Phe_NDVI_, Phe_plant_ was characterized by later green-up, earlier senescence, and a shorter growing season, suggesting that dominant species may optimize their growth timing to avoid unfavorable spring and autumn conditions. Site-level comparisons revealed that Phe_GPP_ and Phe_NDVI_ estimates were relatively consistent at HBS, with SOS and EOS from Phe_GPP_ slightly earlier than those from Phe_NDVI_. In contrast, at CBF, Phe_GPP_ showed an earlier SOS, delayed EOS, and longer GSL than Phe_NDVI_. This discrepancy is attributable to the complex, multi-layered canopy structure of this mixed forest, wherein the photosynthetic activity of cold-tolerant evergreen conifers (*Pinus koraiensis*) and the phenology of understory species—both captured more directly by GPP—are partially masked in NDVI data by the overstory deciduous canopy (e.g., *Quercus mongolica*). A key finding of this comparative study is that the five extraction methods (GST, DLT, DTC, STC, SSC) produced significant variability in phenological metrics, especially for EOS and GSL. Specifically, compared to curvature-based methods (DTC, STC, SSC), threshold-based methods (DLT, GST) not only provided more consistent cross-source estimates but also generally yielded later SOS, earlier EOS, and consequently a shorter GSL. Estimates of Phe_GPP_ and Phe_NDVI_ derived using the same non-threshold method diverged considerably, especially for EOS and GSL. Strikingly, at CBF, negative correlations were detected among the derived EOS or GSL values. These findings underscore the critical need to select appropriate phenological types and extraction methods for the reliable characterization of phenological shifts, particularly for EOS. Therefore, to reliably characterize phenological shifts—especially for the highly variable EOS—future studies must carefully select among data fitting and determination methods, with explicit consideration of vegetation composition and understory dynamics in complex ecosystems.

## Data Availability

The data presented in this study are available on request from the corresponding author. The original plant phenology records can be accessed through the data service system of CERN (http://www.cnern.org.cn/data/initDRsearch?classcode=STA accessed on 26 August 2025). The DOI for the original flux data of CBF and HBS is https://doi.org/10.11922/csdata.2020.0041.zh and https://doi.org/10.11922/csdata.2020.0034.zh, respectively.
